# Regional expression of myocardial sheet dysfunction in dystrophin-deficient cardiomyopathy elucidated with diffusion tensor MRI and optical calcium mapping

**DOI:** 10.1186/1532-429X-14-S1-O31

**Published:** 2012-02-01

**Authors:** Ya-Jian Cheng, Di Lang, Shelton D Caruthers, Gregory Lanza, Igor Efimov, Junjie Chen, Samuel A Wickline

**Affiliations:** 1 Biomedical Engineering, Washington University in Saint Louis, Saint Louis, MO, USA; 2 Cardiovascular Division, Washington University in Saint Louis, School of Medicine, Saint Louis, MO, USA

## Summary

Duchene Muscular Dystrophy (DMD) is a lethal disease caused by ubiquitous lack of dystrophin, but the interaction with regional cardiac mechanical forces that may facilitate eventual expression of abnormal contractile function is unknown. Diffusion tensor MRI (DTI) was used to evaluate function in Langendorff perfused hearts in the mdx mouse model of DMD. Abnormal calcium kinetics (by optical mapping) and sheet mechanics (by DTMRI) occurred more prominently at the mid-upper ventricle, suggesting that regional mechanics influence the development of heart failure.

## Background

The relationship between abnormal sarcoplasmic Ca2+ handling and cardiac structural mechanics in mdx mice has not been defined. DTI has been used to quantify the contribution of myocardial sheet architecture in ventricular wall thickening mechanics. In this study, DTI was employed to assess altered sheet mechanics that might be influenced by impaired calcium homeostasis. Furthermore, optical mapping of AP and CaT were employed to characterize local difference in ex vivo beating hearts.

## Methods

Sixteen-month old mdx (n = 10) and age matched wildtype (WT, n = 10) mouse hearts were prepared for Langendorff perfusion by sequentially cardiac arrest in diastole and systole for DTI: The first group (WT-NC & mdx-NC) was perfused with regular St. Thomas’ cardioplegic solution containing normal [Ca2+] (1.2 mM) to arrest hearts in diastole. To assess the regulatory effect of [Ca2+] on diastolic sheet mechanics, the second group (WT-LC & mdx-LC) was perfused with modified cardioplegic solution containing low [Ca2+] (0.078 mM). All hearts were then reperfused with Krebs buffer to resume beating followed by 2.5mM barium-induced systolic arrest for DTI. Absolute values of sheet angles, |β|s, in each heart were calculated from the diffusion tensors. MANOVA was used for statistical analysis. Another set of Langendorff beating mouse hearts from WT (n=2) and mdx (n=3) mice were optically mapped. RH237 and Rhod-2 AM were used as membrane potential and calcium probes. Steady state restitution pacing protocol was applied to estimate the calcium dynamics at different heart rates.

## Results

**Figure 1:**
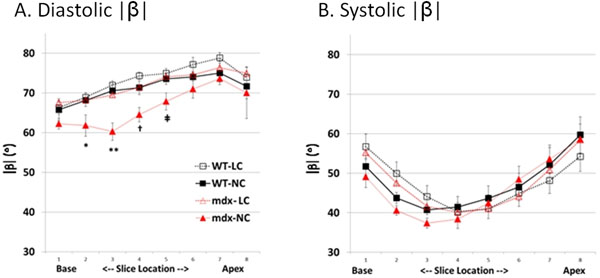
DTI measured |β| from base to apex in diastole (A) and systole (B). Normal calcium concentration perfused mdx heart (mdx-NC) exhibit lower |β| than all other three groups at mid-upper ventricle. Surprisingly, while mdx hearts were perfused with low calcium concentration (mdx-LC), the |β| was restored to normal level. *, p =0.04; **, p=0.001; †, p = 0.003; ++, p < 0.02. Data are shown as mean ± SEM. N = 5 for each group.

**Figure 2:**
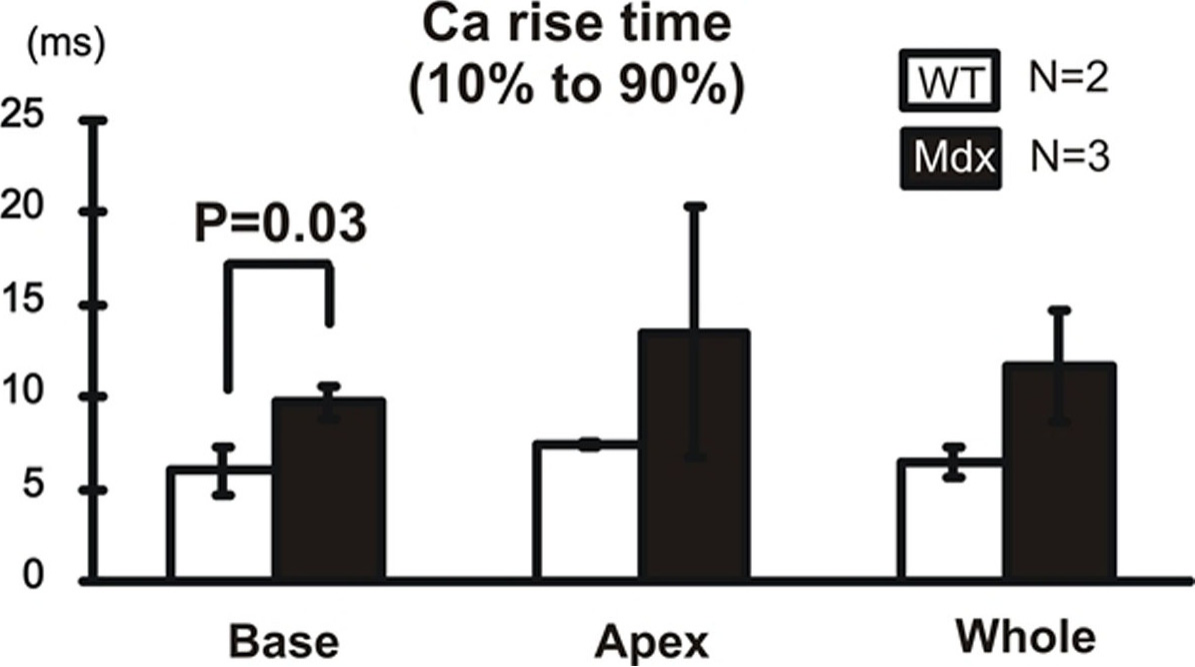
Calcium transient rise time from 10% to 90% amplitude based on optical mapping signals in both WT and mdx mice. The signals from base area, apex area and the whole ventricle were statistically compared. Significantly longer rise time in mdx mice ventricle base was observed comparing to WT group.

In diastole, mdx hearts exhibited lower |β| in mid-upper ventricle than WT did. Reducing [Ca2+] in cardioplegic solution normalized the diastolic |β| in mdx hearts but had no detectable effect on WT hearts. No significant difference of systolic |β| was observed between mdx and WT hearts. (Fig. 1) Optical mapping of CaT showed a localized Ca alternans and Ca rising time (Fig. 2) abnormality in mdx mice’s mid-upper ventricle.

## Conclusions

The observed lower diastolic |β| in mdx hearts indicates that mdx cardiomyocytes fail to fully relax in diastole. The regulatory effect of [Ca2+] on diastolic |β| of mdx hearts suggests that the myocardial sheet diastolic dysfunction is reversible despite the advance age of mdx mice. Optical mapping of CaT in beating hearts further confirmed the DTI-detected localized calcium-dependent abnormality, indicating that the ubiquitous dystrophin deficiency in mdx mice depends on regional cardiac features for its affection. In conclusion, the disturbed sheet mechanics may reflect functional consequences of abnormal Ca2+ handling in vivo, which likely correlates with membrane calcium channel dysfunction that precedes eventual cardiac failure.

## Funding

RO1 HL073646.

